# Change in cost and affordability of a typical and nutritionally adequate diet among socio-economic groups in rural Nepal after the 2008 food price crisis

**DOI:** 10.1007/s12571-018-0799-y

**Published:** 2018-05-12

**Authors:** Nasima Akhter, Naomi Saville, Bhim Shrestha, Dharma S. Manandhar, David Osrin, Anthony Costello, Andrew Seal

**Affiliations:** 1 UCL Institute for Global Health, 30 Guilford Street, London, WC1N 1EH UK; 2 Wolfson Research Institute for Health and Wellbeing, Wolfson Building, Durham University Queen’s Campus, University Boulevard, Stockton on Tees, Thornaby, TS17 6BH UK; 3 Mother and Infant Research Activities, YB Bhawan, Thapathali, Kathmandu, 921 Nepal

**Keywords:** Food price crisis, Nutritionally Adequate Diet, Typical food basket, Poverty, Food security, Malnutrition

## Abstract

Diet quality is an important determinant of nutrition and food security and access can be constrained by changes in food prices and affordability. Poverty, malnutrition, and food insecurity are high in Nepal and may have been aggravated by the 2008 food price crisis. To assess the potential impact of the food price crisis on the affordability of a nutritionally adequate diet in the rural plains of Nepal, data on consumption patterns and local food prices were used to construct typical food baskets, consumed by four different wealth groups in Dhanusha district in 2005 and 2008. A modelled diet designed to meet household requirements for energy and essential nutrients at minimum cost, was also constructed using the ‘Cost of Diet’ linear programming tool, developed by Save the Children. Between 2005 and 2008, the cost of the four typical food baskets increased by 19% – 26% and the cost of the nutritionally adequate modelled diet increased by 28%. Typical food baskets of all wealth groups were low in macro and micronutrients. Income data for the four wealth groups in 2005 and 2008 were used to assess diet affordability. The nutritionally adequate diet was not affordable for poorer households in both 2005 and 2008. Due to an increase in household income levels, the affordability scenario did not deteriorate further in 2008. Poverty constrained access to nutritionally adequate diets for rural households in Dhanusha, even before the 2008 food price crisis. Despite increased income in 2008, households remain financially unable to meet their nutritional requirements.

**Electronic supplementary material:**

The online version of this article (10.1007/s12571-018-0799-y) contains supplementary material, which is available to authorized users.

## Introduction

Although progress has been made in reducing hunger, about 795 million people are still undernourished globally, of whom about 780 million live in developing countries and are unable to access enough food for an active and healthy life (Food and Agriculture Organisation 2015). Diet quality is an important determinant of the food and nutrition security of a population and is influenced by food availability, access, utilisation and affordability at both country and household level. Since food cost is the most important determinant of food purchasing decisions (Lo et al. 2009), a food price rise can exacerbate food insecurity and increase the risk of malnutrition (Martin-Prevel et al. 2012). Low diet quality is often associated with poor socio-economic status (SES). Monsivais et al. (2012) observed that the differential amounts spent among households of different socio-economic backgrounds explained the variable quality of diet. Similarly, a modelling study of diets for French adults found that imposing cost constraints led to a diet plan lower in vitamin C and β–carotene than that of the average population (Darmon et al. 2002). Food prices and income determine purchasing power and the affordability of healthier foods (Drewnowski and Darmon 2005; Darmon and Drewnowski 2008) and may suggest how resilient or vulnerable households can be in responding to a food price crisis. However, the relationship between food price and income is not always predictable, as the larger food environment, i.e. availability, convenience, and desirability of foods in a certain location, can also play important roles (Herforth and Ahmed 2015). It is therefore important to understand and examine the impact of food prices in a given context.

The 2008 food price crisis increased the susceptibility of many vulnerable households to malnutrition, but the effect may have varied around the world due to many factors, including regional variability in price rises, household income levels, consumption patterns, and preferences for available food items (Levine 2012; Mahajan et al. 2015). The World Bank estimated that, globally, food price increases caused an extra 44 million people to be undernourished and 100 million people to fall into poverty (World Bank 2008). Price rises affected the nutritional quality of diets, especially for the lowest income countries and poorer socio-economic groups within a country (Green et al. 2013; Anríquez et al. 2013; Mahajan et al. 2015). One reason for this is that the prices of nutrient-dense food items rose disproportionately and access to higher quality nutritious foods became more restricted among poorer households. Analysis of national level data may mask these differences. In Seattle, USA, the trend in food prices between 2004 and 2008 showed that when foods were grouped by their nutrient density, inflation for the highest quintile was nearly double that of the lowest (Monsivais et al. 2010). In Ghana, the national impact of the 2008 global food price crisis was moderate. However, impact varied by region due to differences in consumption practices and income levels. Poorer households in urban areas who bought most of their food, and those who lived in northern Ghana and spent a large proportion of their income on food, were the worst affected (Cudjoe et al. 2010). Correct assessment of the nutritional impact of a crisis can be difficult, as there is often a lack of geographically detailed price data and methodologically sound studies (Benson et al. 2013; Gibson 2013). To guide program planning and suggest appropriate policy responses in a local context, it is important to understand the extent of local food price inflation and the heterogeneity of the population impacts (Mahajan et al. 2015).

Various measurement tools have been used to understand the differential impact of food prices on food security and nutrition in different regions and among households with varying socio-economic status (Anríquez et al. 2013; Green et al. 2013; Akter and Basher 2014). Economic analysis has been done to measure how the demand for different food groups responds to changes in price (Green et al. 2013). Some studies have focused only on staple prices, whereas price changes could vary for different food groups, and this needs to be considered in assessing the potential nutritional impact of changes in purchasing power (Nordström and Thunström 2011). A food price index based on the prices of items in a typical food basket from a country or region can be used to estimate food price inflation and indicate the magnitude of loss of household purchasing power (Levine 2012). Using data from 35 countries, the World Food Programme estimated that, on average, the cost of a food basket increased by 36% between 2007 and 2008 (Brinkman et al. 2010). In this WFP study, the cost of a food basket in Nepal (for rural and urban areas combined) increased by 19% over one year between 2007 and 2008. However, the study did not assess whether household income had changed or food substitution had occurred.

A food price index typically only takes into account the energy sufficiency of the food basket, rather than its full nutritional adequacy. It is generally made at country or regional level and does not account for household-level socio-economic differences. In addition, a change in food prices needs to be examined in relation to change in income levels to assess potential effects on different groups (Mahajan et al. 2015). The importance of income data was apparent from an analysis in Sri Lanka, which found a 55% increase in food prices, a 26% increase in price of non-food items, but a 57% increase in income between 2007 and 2009. The undernourished proportion of the population was expected to increase by 1.7%, but the predicted increase was much higher when changes in income were not taken into account (Korale-Gedara et al. 2012).

Estimation of the cost of a nutritionally adequate diet using linear programming and household income levels can help model the impact of a food price rise on the affordability of a nutritionally adequate diet and thereby indicate its potential nutritional impact. In several countries, linear programming has been used to plan and estimate the cost of a nutritionally adequate diet for children, men, and women (Briend et al. 2001; Darmon et al. 2002, 2006; Rambeloson et al. 2008; Dibari et al. 2012). The ‘Cost of Diet (CoD)’ tool was developed by Save the Children to help design diets for the whole household (Save the Children UK 2011). The tool can be used to estimate the cost and affordability of a household diet, which can be helpful in understanding the potential for a localized nutritional impact of a crisis among households with different socio-economic backgrounds (Save the Children UK 2009a). Several recent studies have used CoD (Save the Children UK 2009a, b; Frega et al. 2012; Baldi et al. 2013; Save the Children UK 2013a; Geniez et al. 2014; Termote et al. 2014; Biehl et al. 2016), reflecting that it can be used as an advocacy tool to promote food-based interventions or social safety net programmes, depending on the specific context.

In this study, we used local market price data from the rural plains of Nepal before and during the global food price crisis, to estimate the cost, affordability, and nutritional content of typical food baskets (TFB) and a modelled, minimum-cost, nutritionally adequate diet (NAD). The unique features of this study are that, rather than using country or regional level food baskets, we calculated food price inflation based on a local food price index and utilised consumption pattern and income estimates from the area to plan the TFB as well as the modelled NAD. These analyses, although they do not compare regions, are important for Nepal as Shively et al. (2015) found that the relationship between environmental conditions and nutritional status of children varied in different regions. Furthermore, these analyses of impact of price rise on affordability scenarios for the two methods (TFB and NAD) were disaggregated by SES groups, which enabled our study to examine the hypothesis that after adjusting for changes in income, the potential dietary impact of the 2008 global food price crisis would vary by socio-economic group.

## Methods

Nepal is a diverse country with varied geography, topography, and related agricultural and consumption practices. It has three ecological zones: Hills, Mountains, and Plains. The Plains occupy the smallest area but have the largest population. The study was conducted in Dhanusha district, which lies in the Plains bordering India and has an area of 1180 Sq. Km (Central Bureau of Statistics 2008). It ranks 43rd out of 75 Districts on the Human Development Index (Programme 2009).

The study categorized the population of Dhanusha into four socio-economic groups (wealth groups) and determined a Typical Food Basket (TFB) for each group, which is detailed in later sections. The TFB included a fixed set of food items and met the energy (kcal) requirements of household members. A minimum-cost, Nutritionally Adequate Diet (NAD) that met the requirements for energy and key nutrients for households in all wealth groups was also formulated using linear programming. We estimated the cost of the TFB and NAD and assessed the affordability for each wealth group, before and after the 2008 global food price crisis.

### Data sources and analysis

We combined data collected in Dhanusha, during several studies, by enumerators from Mother and Infant Research Activities (MIRA), a non-governmental organization. These were a household economy approach (HEA) study, data from a household surveillance system (HSS) study, a survey of market prices, and a survey of change in income by sources. Informed consent was obtained from all individual participants included in the study.

Initial data were collected during March–June 2006 in 60 administrative units, called Village Development Committees (VDC) in Dhanusha, using the HEA method introduced by Save the Children UK (Holzmann et al. 2008). The HEA uses participatory group interviews to assess food security and the actual or predicted impact of a livelihood shock in an area. The HEA data from Dhanusha provided a description of the characteristics of wealth groups (Very poor, Poor, Middle, Better-off) and their distribution within each of the 60 VDC. It described their livelihoods and food security patterns, including the annual consumption of food items and income and expenditure by source for the year 2005 (Akhter 2013). The HEA provided useful data on sources of income and the estimated proportion of cash of food-derived income for each wealth group, but quantification of average income varied and was less reliable. Hence, median expenditure data was determined and considered a reliable proxy estimate for income for each wealth group (Table 1).

**Table 1:** Indicators used and sources of data: Household Economy Approach 2006 study

Instrument	Data type	Respondents	Data collected	Outcome Indicators
Household Economy Approach (HEA)	Livelihoods	Wealth group representatives	Ownership of assets, livelihoods, coping mechanisms	A narrative description of wealth groups and their proportions within each VDC
	Expenditure	Wealth group representatives	Total expenditure by category	Median daily household expenditure
	Income	Wealth group representatives	Source of income for wealth groups, as food derived and cash income	% of total income by categories (food derived, cash income) by Wealth groups % contribution of income sources to total cash income by Wealth groups
	Food consumption	Wealth group representatives	Name and amount consumed annually for individual foods within each food group	The most frequently consumed food item by each wealth group
	2005 Price of food items	Vendor/s at the main local market/VDC	Prices of commonly consumed food items	Mean price (Nepalese Rupees)/food item

**Table 2:** Indicators used and sources of data: Household Surveillance Systems and others

Instrument	Data type	Respondents	Data utilised	Outcome Indicators
Household Surveillance System (HSS)	Assets and housing characteristics	Household representative	Ownership of assets, type of roof and walls of house, primary source of staple food	A ranking of households using an asset index derived by Principal Component Analysis (PCA)
HSS	Dietary intake	Household representative	Consumption from food groups in the last 24 h (Food Frequency Questionnaire)	Percentage household-level consumption of food groups by wealth group
2008 Change in Income Study	Change in income by source	Key Informant for income sources	Pay rate/monthly salary by source of income	% change in income source; % change in income by category; % change in total income by wealth group
2008 Food Price Survey	Price of food items	Vendor/s at local markets	Mean prices of commonly consumed food items	Mean price (Nepalese Rupees) of foods
Cost of Diet software	Nutritionally adequate diets optimised for minimum cost using linear programming		*Mean price of food items in 2005 and 2008 *Physical Activity level of household members, median income / wealth group in 2005 and 2008	Minimum cost of a nutritionally adequate diet for Dhanusha households in 2005 and 2008, and affordability by wealth groups

*Cost of Diet utilised these data gathered by other means

Baseline data (mid-September 2006 to mid-April 2007) from a household surveillance system (HSS), on household assets, housing characteristics, and primary sources of staple food (Table 2) were analysed using principal components analysis to create a SES index and rank households from the same 60 VDC. In each of the VDC, the proportion of households estimated to belong in each wealth group was available from HEA data and used to group the ranked households into ‘Very Poor’, ‘Poor’, ‘Middle’, and ‘Better-off’ wealth groups, in each VDC so that the SES characteristics of the wealth groups could be described.

In 2008, we collected data on changes in income levels. Firstly, HEA data were used to create income profiles for each wealth group, which detailed their sources of income (e.g. income from daily labour, factory work, self-employment such as in a grocery shop, salaried workers, and seasonal migratory work within Nepal and abroad, and Government staff salaries). These were used as a basis for calculating income change. Key informants who were engaged in cash-income activities such as. daily waged labour and self-employment were identified by wealth groups and interviewed about current (2008) and recalled (2005) income levels (Akhter 2013). Using income profiles, data were collected from all VDCs for change in income for the commonly used income categories (e.g. agricultural labour daily pay: *n* = 60, one per VDC; seasonal migratory income: total *n* = 55; overseas income from Arab countries: *n* = 57; Malaysia = 59). Other income data were collected through two key informant interviews per six MIRA unit offices, i.e. maximum of 12 interviews/ income category (e.g. earnings from self-employment such as a snack shop = 10, medicine shop = 11). The monthly salaries of government employees were collected from the District Development Committee office, District Public Health Office and District Education office. The income data was used to calculate the percentage change in income between the two periods, and the change was then used to estimate 2008 income levels. Using median total expenditure as a proxy for income in 2005, we modelled 2008 income data as:

$$ {\displaystyle \begin{array}{l}\mathrm{Cash}\ \mathrm{income}\kern0.75em =\left(2005\kern0.5em cash\kern0.5em in com\mathrm{e}\right)+\left[\left(2005\kern0.5em cash\kern0.5em in com e\right)\times \% change\kern0.5em in\kern0.5em cash\kern0.5em in com e\right]\\ {}\mathrm{Food}\ \mathrm{derived}\ \mathrm{income}\kern0.5em =\kern0.5em \left(2005\kern0.5em food\ derived\ income\right)+\Big[\left(2005\kern0.5em food\ derived\ income\right)X\kern0.5em food\\ {} price\kern0.5em in flation\kern0.5em between\kern0.5em 2005\kern0.5em \mathrm{and}\kern0.5em 2008\Big]\;\end{array}} $$

More details of the 2008 income change data collection are included in **Appendix 5**.

HSS data were collected on the consumption of food groups (cereals, roots and tubers, green and other vegetables, fruits, dairy, fish, meat, eggs, pulses and nuts, sugar and honey, and others, such as spices, tea, or coffee) in the last 24 h were summarised and used to define the dietary intake pattern for each wealth group (Akhter 2013). Food price inflation was estimated using price data collected from retail vendors in the most accessible market in each VDC, in 2005 and 2008 (Sep-Oct). Data were collected from the same VDC markets in both years. Allowing for changes (e.g. closure of markets) between 2005 and 2008, food prices from 48 VDCs (one market / VDC) were utilised. Our study did not adjust for possible difference between farm-gate prices and local market prices, but assumed that since data were collected from the most accessible local markets the differences would not be large. Details of this estimation method are presented elsewhere (Akhter 2013).

Data management and analyses were done in the Statistical Package for the Social Sciences (SPSS), version 16.0 (Statistical Package for the Social Sciences 2007), except for linear programming analysis, which was performed using the CoD application (Save the Children 2011; Save the Children 2013a) run using the Solver add-in function in MS-Excel 2003 (Excel 2003). Details of data collection and management are presented elsewhere (Akhter 2013).

### Calculation of parameters used for Typical Food Baskets (TFBs) and Nutritionally Adequate Diet (NAD)

#### Mean food prices

For both 2005 and 2008, after excluding outliers that were either < Q1–1.5*IQR or > Q3 + 1.5*IQR (where Q1 = lower quartile; Q3 = Upper quartile; IQR = Inter Quartile Range) (UK Office for National Statistics 2007), the mean prices of food for each 100 g of purchased item (**Web** Table 1) were used to estimate the cost of TFB and NAD.

#### Median household expenditure

Using HEA data, annual household expenditures by wealth group for 2005 were estimated. Median expenditure levels served as a proxy for income (Akhter 2013).

#### Income level for wealth groups in 2008

The income levels of wealth groups in 2008 were estimated using 2005 expenditure as the base and incorporating changes in income between 2005 and 2008. Details of these calculations are mentioned above (Akhter 2013).

#### Food composition Tables

A food composition database for 64 commonly available items in Dhanusha was prepared using the USDA National Nutrient database (U.S. Department of Agriculture 2009), the East Asia Food Composition database (Food and Agriculture Organisation 1972) and the Bangladeshi food composition tables (HKI 1988). These food values were inputted into the Cost of Diet (CoD) database (Save the Children UK 2011) and were also used for estimation of the food energy (kcal) in the TFB.

#### Household demographics and energy requirements

The demographics of a model household, and the energy and nutrient requirements of its members were estimated on the basis of primary data and relevant findings from national and regional studies (Hirai et al. 1993; Food and Agriculture Organisation 2010; Ministry of Health and Population (MoHP) 2012). Firstly, based on our HEA findings a model household was defined as including six members: 2 boys (aged 2–3 and 5–6 years), 1 adolescent girl of 13–14, and 3 adults (1 male aged 37, 1 female aged 28, and 1 female aged 45–50) (Akhter 2013). Secondly, the energy requirements of household members were set based on FAO standards and available evidence about physical activity levels (PAL) for Nepal (Sudo et al. 2006; Food and Agriculture Organisation 2004a). For the children and adolescents, habitual activity levels recommended by the Food and Agriculture Organisation (2004a) were used. Both adult men and women aged 20–44 were considered to be moderately active (Food and Agriculture Organisation 2004a; Sudo et al. 2006; Central Bureau of Statistics 2008). The senior woman in the household, aged 45–50, was considered to be lightly active (Akhter 2013).

The PAL and corresponding energy requirements of the household members for both TFB and NAD were calculated using guidelines developed by the joint FAO/WHO/UNU Expert Consultation in 2001 (Food and Agriculture Organisation 2004b). In addition, the CoD program used in-built requirements set for macro- and micronutrients specific for age, sex, and pregnancy or lactation (Save the Children UK 2013a); (Frega et al. 2012).

The same six members and their age, sex, physiological status, and activity levels were used in the calculation of TFB and NAD. Because there was a paucity of data on whether body size or PAL varied in rural Nepal by wealth status, for the purposes of the analysis we assumed that the energy requirements of households did not vary by wealth group. We considered that, although members of a wealthier household would be heavier than members of a poorer household, the PAL would be lower for wealthier than for poorer households and the energy needs were therefore likely to be approximately equal across wealth groups.

### Estimating the cost of household Typical Food Baskets (TFB)

To estimate the cost of a TFB, a daily food basket for an adult male was planned and costed for each wealth group. The weight of each food group to be included was first estimated for the combined wealth groups using data from another study (Hirai et al. 1993). Secondly, this food basket was then adjusted to create 4 wealth group specific food baskets using our HSS 24-h data on food group consumption (Table 3). Thirdly, for each wealth group, the most frequently consumed item/s in a food group (based on HEA data) was selected for inclusion (Akhter 2013). The energy content of the included food items met the requirement for an adult male.

**Table 3:** Food items included in the typical daily household food baskets, by wealth group, Dhanusha, Nepal

	Very poor		Poor		Middle		Better-off	
Food groups	Item	Amount (g)	Item	Amount (g)	Item	Amount (g)	Item	Amount (g)
Cereals	Rice, parboiled	2331	Rice, parboiled	2138	Rice, fine grain	2076	Rice, fine grain	1703
	Whole wheat flour	278	Whole wheat flour	255	Whole wheat flour	244	Wheat flour, fine	188
Roots/ tubers	Potato	582	Potato	631	Potato	651	Potato	664
Coloured vegetables	Red amaranth	346	Red amaranth	382	Red amaranth	409	Red amaranth	440
Other vegetables	Eggplant	279	Eggplant	309	Eggplant	333	Pointed gourd	372
Pulses	Kheshari lentil (grass pea)	105	Red lentil	148	Red lentil	162	Yellow split pea	175
Oil	Mustard oil	112	Mustard oil	124	Mustard oil	121	Mustard oil	120
Dairy	Cow milk	359	Cow milk	999	Cow milk	1553	Cow milk	2049
Meat	–	–	–	–	–	–	Chicken, broiler	77
Fish	Mixed small fishes	62	Mixed small fishes	65	Fish, Silver carp	51	Fish, Silver carp	49
Others	Yellow mustard	62	Yellow mustard	69	Yellow mustard	75	Yellow mustard	79
Sugar	–	–	–	–	–	–	Sugar	216
Fruit	–	–	–	–	–	–	Apple	90

Finally, to estimate the cost of the household TFB, the cost of the food basket for the one adult male was calculated using market price data. This cost (in Nepalese Rupees, per kcal), was then multiplied by the total energy requirement for all household members to give the cost of the household TFB. This process was repeated for each wealth group. The 2005 and 2008 TFB included the same set of food items.

### Modelling a minimum-cost, nutritionally adequate diet (NAD)

The Cost of Diet (CoD) tool was run to select the foods and the amount of each food that would meet household requirements for energy (kcal) and nutrients with the objective function set to minimize cost. The CoD generated the lowest cost diet plan for the household that was nutritionally adequate. One NAD was formulated for all four wealth groups.

Nutrient constraints were set so that the NAD would: provide an energy content equal to the sum of the requirements of all household members; provide 30% of household energy requirements from fats; provide at least 100% of the recommended intakes of vitamin A, vitamin C, thiamine, riboflavin, niacin, vitamin B6, folic acid, vitamin B12, pantothenic acid, calcium, iron, and zinc (Food and Agriculture Organisation 2004a, b); and would not exceed the recommended safe intake levels for vitamin A, iron, vitamin C, calcium and niacin (Food and Agriculture Organisation 2004a; Save the Children UK 2011).

Food consumption constraints comprised intake limits set for food groups and food items. Food group constraints were set as the minimum and maximum allowable intake from a particular food group per week, based on HSS 24-h dietary recall data, published findings, and anecdotal evidence (Hirai et al. 1993; Food and Agriculture Organisation 2004a). Constraints for food items were defined as the allowable number of portions per person per week. They were all set between 0 and 21 portions per person per week. The average intake of food consumed per meal by a 12–23 month child was considered a reference portion size and the CoD adjusted to the size for each member in relation to their energy requirements Portion sizes for different food types were taken from the CoD manual, when available; if not, the portion size for a similar food type was used (Save the Children UK 2011).

## Results

### Characteristics of wealth groups

HEA interviews indicated that 25.9%, 31.7%, 33.0%, and 8.5% households in Dhanusha were from Very Poor, Poor, Middle, and Better-off wealth groups, respectively. Livelihoods were dominated by agriculture. Poorer households (Very Poor and Poor) were mostly landless or owned a small amount of land and had limited food production. They relied on agricultural daily-waged labour, paid in cash or grain, or other unstable sources of income. The Middle and Better-off were farming households and had earnings from agricultural production, regular jobs, and overseas remittances (**Web** Table 2). Median annual household expenditure (a proxy for income) showed a gradient and was almost four times higher among Better-off than Very Poor households (**Web** Table 2). The percentage of total expenditure on food in 2005 also varied by wealth group (Very Poor 58%; Poor 45%; Middle 32%; Better-off 24%).

### Cost and nutritional adequacy of Typical Food Baskets (TFB)

**Fig. 1:**
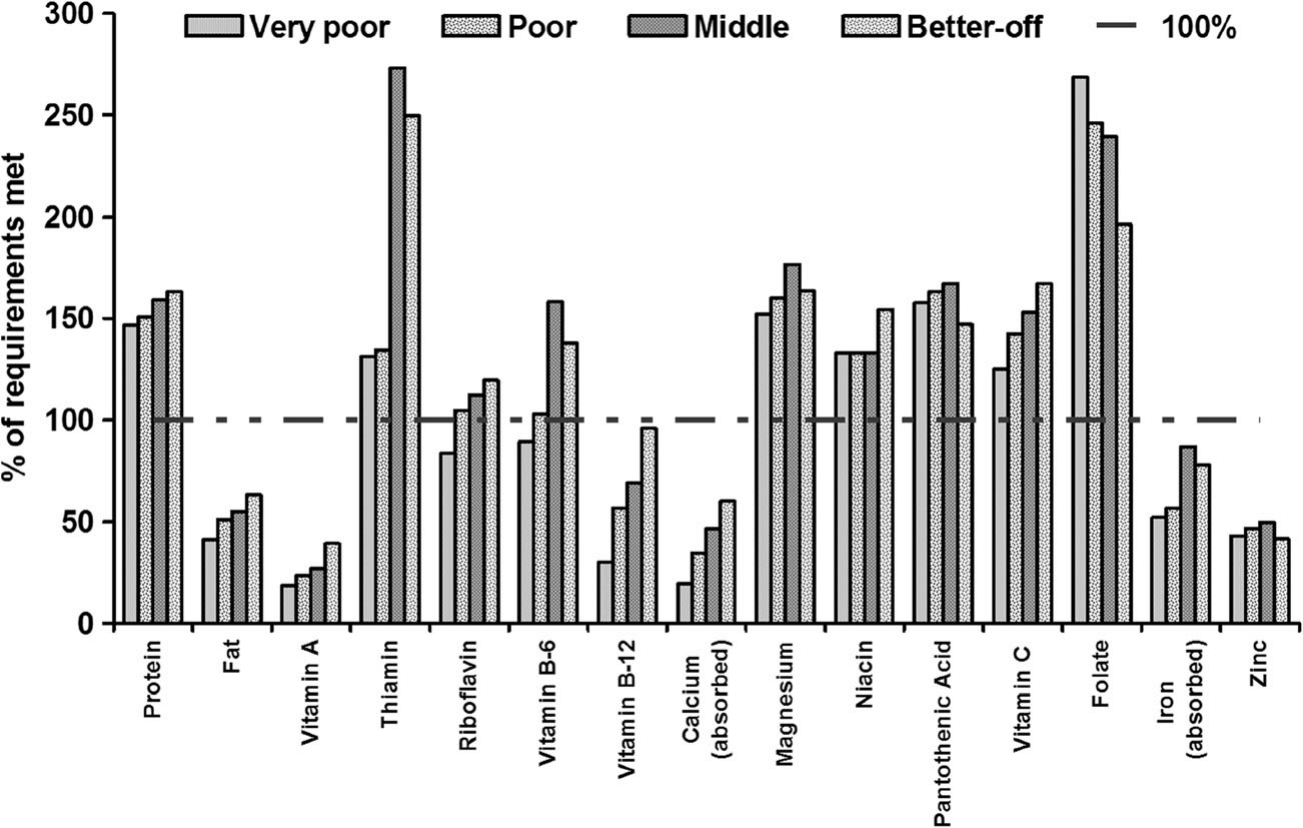
Nutrient content of a typical daily household food basket, by wealth groups, Dhanusha, Nepal

TFB for all wealth groups were assumed to remain constant between 2005 and 2008. They included rice, wheat, potatoes, vegetables, pulses, dairy, and oil, but were more diverse among Better-off households (Table 3). Following the 2008 food price crisis, the absolute costs of the TFB of Very Poor, Poor, Middle, and Better-off households increased by 19.2%, 22.0%, 26.1%, and 23.4%, respectively (Table 4). For both periods, the cost of the TFB for Better-off households was much higher than for poorer households, but the proportion of their income spent on the TFB was much lower. Between 2005 and 2008 the proportion of income spent on food fell for all wealth groups, but the decrease in the proportion of income spent on a TFB was greatest among poorer households (Very Poor, −6.5%; Poor, −3.1%, Middle, −0.3%; Better-off, −1.1%). However, the TFBs of all wealth groups were low in several nutrients, including vitamin A, fats, vitamin B12, calcium, iron, and zinc (Fig. 1). The food basket for the Very Poor was also deficient in riboflavin and vitamin B6.

**Table 4:** Cost of a typical daily household food basket in 2005 and 2008, by wealth group, Dhanusha, Nepal

Wealth group	2005		2008	
	Cost (Nepalese Rupees)^**a**^	% of HH Income	Cost (Nepalese Rupees)	% of HH Income
Very Poor	96.5	75.2	115.0	68.7
Poor	113.7	64.0	138.7	60.9
Middle	127.4	39.5	160.7	39.2
Better-off	167.5	30.3	206.7	29.2

Typical household with 6 members: boy (2–3 years), boy (5–6 years), adolescent girl (13–14 years),

adult male (37 years), adult female (28 years), adult female (45–50 years)

^a^1 US$ = 66.5 Nepalese Rupees in 2005, 73.2 Nepalese Rupees in 2008

### Cost and affordability of a Nutritionally Adequate Diet (NAD)

**Fig. 2:**
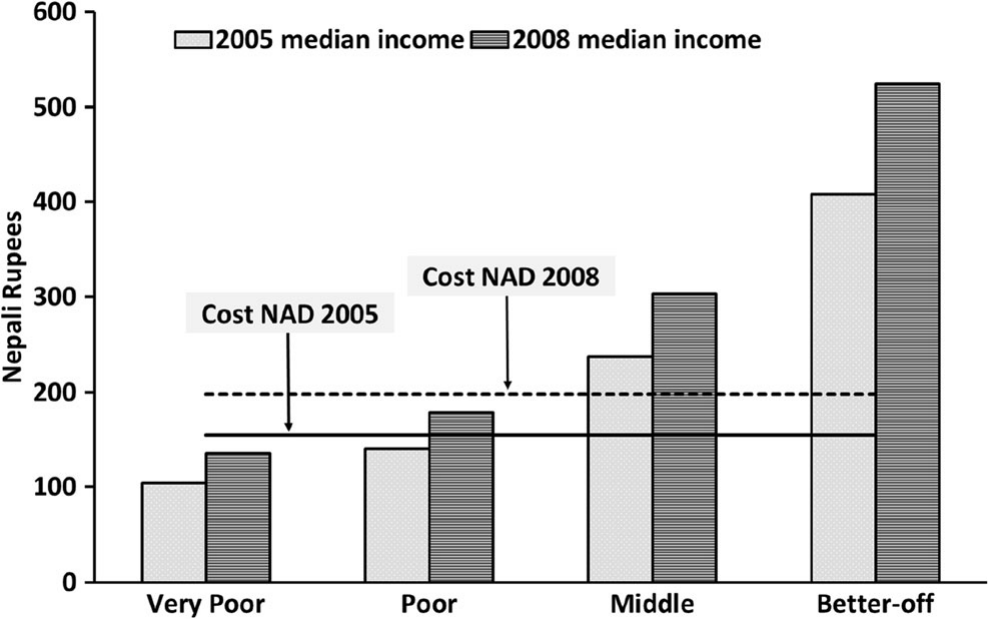
Affordability of a minimum-cost, nutritionally adequate diet in 2005 and 2008, by wealth groups, Dhanusha, Nepal

The CoD linear programming tool generated a minimum cost NAD for the model household, and the cost was calculated for 2005 and 2008 (Table 5). In both years, the NAD met or exceeded the nutritional requirements (Web Table 4) of household members. However, in 2005 neither Very Poor nor Poor households were able to afford a NAD **(**Fig. 2**)**. In 2008, the cost of the NAD increased by 28% over the 2005 cost, but the incomes of Very Poor, Poor, Middle and Better-off households also increased by 32%, 27%, 25% and 28%, respectively. After the election of the Nepal constituent assembly in 2008, a political decision was taken to increase government salaries with a bias towards the most poorly paid and to establish a minimum wage for agricultural and factory workers (Ministry of Labour and Transport 2008 Nov 18) **(**Fig. 2**)**. Despite this increase in income, Very Poor and Poor households were still unable to afford a NAD in 2008 **(**Fig. 2**)**. To afford a NAD, poorer households would need to spend more than their total income on food, whereas Better-off households would need to spend roughly one-third (% income required to purchase a NAD in 2005 and, 2008 for Very poor: 148%, 147%; Poor: 110%, 111%; Middle: 65%, 65%; Better-off: 38%, 38%, respectively).

**Table 5:** Content and cost of a nutritionally adequate daily diet for a typical household in Dhanusha in 2005 and 2008, calculated using linear programming

	2005		2008	
Food Item	Quantity (g)	Cost (Nepalese Rupees)	Quantity (g)	Cost (Nepalese Rupees)
Rice, Parboiled	1456	29.0	1607	36.5
Rice, fine grain (Mansuli)	353	7.6	202	5.6
Cow Milk	1621	38.1	1695	45.2
Fish, Rahu	267	30.2	277	45.3
Mustard oil	46	4.4	46	6.2
Horse gram	139	4.4	139	6.6
Potato	387	4.6	387	5.0
Cumin	5	0.9	6	1.5
Chilli powder	33	2.6	33	4.0
GLV^a^, Amaranth	975	9.2	–	–
GLV, Jute leaves	319	4.8	827	13.0
Garlic	65	3.3	65	3.0
Vegetable Ghee	246	15.1	244	25.6
Total cost		154.1		197.8

Typical household with 6 members: Boy (2–3 years), Boy (5–6 years), Adolescent girl (13–14 years),

Adult male (37 years), Adult female (28 years), Adult female (45–50 years)

^a^Green leafy vegetables

## Discussion

We estimated the nutrient content and cost of a TFB, for different wealth groups in a rural district in the plains of Nepal. We estimated the minimum cost of a NAD designed using linear programming and assessed whether the NAD was affordable by different wealth groups, before and after the 2008 global food price crisis.

Although we hypothesised that the food price crisis would have a higher negative nutritional impact on poorer wealth groups, we found that the TFB for all wealth groups did not meet 100% of nutrient requirements. Furthermore, the poorer households could not afford a cost-minimised NAD in both 2005 and 2008. Although the cost of both the TFB and the NAD increased significantly in 2008, the affordability scenario did not deteriorate as an increase in income also occurred. Salaries of government staff in Nepal increased after the Maoist government took over in August 2008, which may have also pushed up payments in other sectors, and a minimum wage rate was established (Ministry of Finance 2009; Akhter 2013). It is important to note that, despite our finding that the price rises were buffered by an immediate increase in incomes, our results indicate that about 57% of households in Dhanusha would have required additional assistance to achieve nutritional adequacy during both periods (Akhter 2013). In addition, when the world food price was falling between 2008 and 2009, there was 19% inflation in food prices in Nepal (Nepal Investment Bank 2009; Food and Agriculture Organisation 2012). Some adverse effects of the price changes may therefore have manifested relatively late in Nepal. The study of Geniez et al. (2014) supports this hypothesis. Using the same tool, they found that 58% of households in the Mountain region and 21% in Kathmandu could not afford a NAD in 2010–2011.

Contrary to our findings, negative impacts of the 2008 food price crisis were seen in a number of countries, especially in low-income countries and low-income groups within them (Ivanic and Martin 2008; Brinkman et al. 2010; Cudjoe et al. 2010; Monsivais et al. 2010; Webb 2010; Mahajan et al. 2015). Several studies have assessed the impact of the 2008 food price crisis using different tools. We used the CoD linear programming tool to formulate a minimum cost, nutritionally adequate household diet and then assessed its affordability. Linear programming has been used more often to model diets at the individual instead of the household level, especially for young children (Briend et al. 2001; Darmon et al. 2002, 2006). Studies have used the CoD software to estimate the cost and affordability of a household diet (Save the Children UK 2009a, 2009b; Frega et al. 2012; Baldi et al. 2013; Save the Children UK 2013b; Geniez et al. 2014) and to advocate promotion of food-based interventions or cash-based social safety net programmes. Consistent with our findings, a least-cost nutritionally adequate diet was unaffordable among poorer households in the pre-crisis period (2005–6) in Ethiopia, Myanmar, and Tanzania (Save the Children UK 2009a). The cost was higher than the average earnings of all wealth groups in Ethiopia, whereas it was higher than the earnings of all ‘very poor’ and some ‘poor’ households in Tanzania and Myanmar (Save the Children 2009a). In Bangladesh, the cost of a least-cost nutritionally adequate diet for 2005/6 and 2007/8 was estimated using the CoD software. The cost increased by 56% and was unaffordable for poorer households in both periods (Save the Children UK 2009b). Estimation of affordability by socio-economic groups in Nepal and other countries highlights the importance of using context-specific data to assess the localized impact of changes in food prices to appropriately guide policy decisions.

Our study found that a typical diet for any wealth group in Dhanusha is still likely to contain less than the recommended intake for several micronutrients, including vitamin A, B12, calcium, iron and zinc; and that the cost of NADs were well above the income levels of poorer households. Although the TFB of Better-off households was more diverse and expensive, it is still low in several micronutrients. Similarly, a study examining the impact of the 2008 food price crisis in Guatemala found disparity in intake among income quintiles, and that households in lower income quintiles were most likely to have diets deficient in vitamin A, B12, folate, and zinc when food prices rise (Iannotti et al. 2012). Assessment of household level dietary diversity using HSS data in Dhanusha (measured by consumption from number of food groups including cereals in the last 24 h) found low diversity among all groups (3.6, 4.0. 4.3 and 4.7 for Very Poor, Poor, Middle and Better-off, respectively) (Akhter 2013). Similar deficiencies were also evident from dietary surveys in rural Nepal (Christian et al. 1998; Parajuli et al. 2012; Ng’eno et al. 2017). A typical food basket generally includes large amounts of rice, providing the bulk of the energy, accompanied by a thin pulse soup, potatoes, and vegetables, with milk as the sole animal origin food (Hirai et al. 1993; Sudo et al. 2006; Parajuli et al. 2012). Ng’eno et al. (2017) examined dietary pattern among socio-economic groups in the plain of Nepal and found that the median daily intake frequencies of most food items were 0 times, but rice, potatoes and vegetable oil were eaten more frequently. Given the low dietary diversity, we only included more than one item per day for cereal and vegetables in the TFB. The number of items per food groups in the TFB was similar to Sudo et al. (2006), which was considered reasonable, based on anecdotal evidence and consultations with field level researchers. We acknowledge that a future study is needed for precise estimation of dietary intake by socio-economic groups, but do not expect that to change our conclusions dramatically.

This lack of diversity in usual diets correlates with the high prevalence of anaemia in the plains of Nepal: 50% in children under five and 42% in women of reproductive age (Ministry of Health and Population (MoHP) 2012). The fact that TFB were nutritionally inadequate and that the CoD could generate a NAD from the same local foods that were affordable by the upper two wealth groups suggests that there could be a gap in nutritional knowledge or behaviour, and that for this segment of the population, communication activities that promote increased consumption of micronutrient-rich foods might be beneficial. Beihl et al. (2016) suggested that improving behaviour can increase dietary diversity and nutrient adequacy in the Nepalese population. Based on our data, we suggest that a combination of approaches, such as food supplementation and dietary fortification, introduction of cash-based social safety nets, and behaviour change approaches are required. The Government of Nepal developed a multi-sector nutrition plan in 2012 which also suggested promotion of a combined approach, using direct and indirect nutrition specific initiatives (e.g. micronutrient supplementation, fortification) along with nutrition sensitive initiatives such as cash and in-kind transfers, school feeding, and nutrition education for various target groups (National Planning Commission 2012).

The main strengths of our study are that detailed, local food price data were collected for 64 commonly consumed items in both 2005 and 2008, which allowed estimation of local level food price inflation (Akhter 2013) and examination of how the affordability of a NAD may have been affected by membership of specific wealth groups in the post-crisis period. We collected food prices from the same market locations in the same season (October–November) in both years. We also assessed the nutritional quality of typical food baskets of wealth groups and calculated the cost of a cost-minimised, nutritionally adequate diet, pre- and post-crisis, allowing for a comparison of affordability.

The study had some limitations. Data were not available on changes in food consumption or expenditure on specific food items and it was not possible to examine substitution effects. Food price data were collected during a festive season when prices tend to peak and we were not able to investigate seasonal variation. However, data were collected for commonly consumed food items and we do not expect seasonal price variation to be large enough to change the results dramatically. Another limitation is that our study estimation used prices from the most accessible rural markets, which may have been slightly higher than farm-gate prices. Also, the prices used were per 100 g of purchased food rather than 100 g of edible serving. However, the poorer households needed to spend more than 100% of their income to afford a NAD, whereas consumption of households’ own produce is likely to only play an important role for Middle and Better-off households. It is therefore unrealistic to assume that the factors would significantly change the main results or conclusions. Due to variation in reported income, we used expenditure data as a proxy for income, an approach which has commonly been done in other studies using this tool (Frega et al. 2012; Baldi et al. 2013). The 2008 income level estimates were not generated from household level data, which may have added some inaccuracy. Nevertheless, the estimation used wealth group-specific income sources and changes in incomes between 2005 and 2008. We think that the changes in income data are realistic as key informants engaged in the specific income sources (e.g. government employees, daily labourers, specific self-employment or other professionals) provided the data. Future studies are required to estimate the cost of an adequate diet during different seasons and to check the acceptability and other factors that may affect adoption of a minimum-cost diet generated using the CoD tool.

## Conclusion

In conclusion, our study found that the typical food baskets of all wealth groups were deficient in micronutrients and the nutritional impact of the food price crisis probably did not vary by wealth group due to an accompanying increase in income. However, the income levels of poorer households, even before the food price crisis, were too low to afford the minimum cost of a nutritionally adequate diet. The inability of poorer households to afford an adequate diet and the inadequacy of nutrients in typical diets indicate that urgent efforts are needed to put in place targeted social safety net programs for poor households, and to provide nutrition education for those households who have the potential to afford nutritional adequacy by making changes to their typical food baskets.

## Electronic supplementary material

ESM 1 (DOCX 85.8 kb)

## Abbreviations

CoD: Cost of Diet
HEA: Household Economy Approach
HSS: Household Surveillance System
VDC: Village Development Committee
PAL: Physical Activity Level
TFB: Typical Food Basket
NAD: Nutritionally Adequate Diet
