# Supplementary material for: Change in cost and affordability of a typical and nutritionally adequate diet among socio-economic groups in rural Nepal after the 2008 food price crisis
Source: Food Secur. 2018 May 12;10(3):615–29. doi: 10.1007/s12571-018-0799-y (PMC6080140; doi:10.1007/s12571-018-0799-y)
Supplement: Supplementary file 1 — (DOCX 85.8 kb) [file 12571_2018_799_MOESM1_ESM.docx]

# Online supplementary materials

# Web Table 1 Food Price Database for 2005 and 2008

| Item | Food Group | Food items | 2005 Price/100g | 2008 Price/100g |
| --- | --- | --- | --- | --- |
| 1 | Cereals | RICE, FINE GRAIN, MANSULI | 2.15 | 2.75 |
| 2 | Cereals | RICE, PARBOILED | 1.99 | 2.27 |
| 3 | Cereals | WHEAT, FLOUR (MAIDA) | 2.45 | 2.90 |
| 4 | Cereals | WHEAT, WHOLE (ATTA) | 1.92 | 2.11 |
| 5 | Dairy | COW MILK | 2.35 | 2.67 |
| 6 | Meat, Egg, Fish | GOAT (INDIA) | 23.21 | 28.07 |
| 7 | Meat, Egg, Fish | CHICKEN, LOCAL | 16.53 | 24.36 |
| 8 | Meat, Egg, Fish | CHICKEN, BROILER | 12.93 | 16.85 |
| 9 | Meat, Egg, Fish | RAHU FISH | 11.31 | 16.36 |
| 10 | Meat, Egg, Fish | COMMON CARP | 11.27 | 15.84 |
| 11 | Meat, Egg, Fish | SILVER CARP | 7.27 | 12.17 |
| 12 | Meat, Egg, Fish | BIG HEAD FISH | 8.85 | 14.13 |
| 13 | Meat, Egg, Fish | MUNGRI, SINGHI | 11.09 | 14.38 |
| 14 | Meat, Egg, Fish | SMALL FISH | 6.13 | 8.01 |
| 15 | Meat, Egg, Fish | GAINCHA, LATTA FISH | 12.13 | 18.20 |
| 16 | Meat, Egg, Fish | CHICKEN EGG, LOCAL | 15.40 | 18.05 |
| 17 | Meat, Egg, Fish | CHICKEN, EGG, FARM | 9.09 | 12.27 |
| 18 | Meat, Egg, Fish | PIGEON | 19.67 | 25.28 |
| 19 | Fat and Oil | MUSTARD OIL | 9.47 | 13.27 |
| 20 | Pulses | YELLOW SPLIT PEA | 5.42 | 7.85 |
| 21 | Pulses | CHICK PEA DAL | 4.85 | 6.68 |
| 22 | Pulses | HORSE GRAM | 3.18 | 4.76 |
| 23 | Pulses | RED LENTIL | 4.50 | 7.52 |
| 24 | Pulses | MUNG DAL | 4.85 | 6.13 |
| 25 | Pulses | BLACK LENTIL | 4.65 | 6.35 |
| 26 | Pulses | KHESHARI YELLOW LENTIL | 3.38 | 3.91 |
| 27 | Roots and Tubers | POTATO | 1.18 | 1.30 |
| 28 | Others | NOODLES | 1.00 | 1.31 |
| 29 | Spices | CORIANDER, WHOLE | 5.58 | 12.35 |
| 30 | Spices | CUMIN | 16.84 | 25.89 |
| 31 | Spices | GINGER | 4.28 | 6.08 |
| 32 | Spices | CHILLI POWDER | 8.04 | 12.27 |
| 33 | Spices | CHILLI, RED, DRIED | 7.03 | 10.57 |
| 34 | Spices | TURMERIC POWDER | 6.60 | 10.32 |
| 35 | Sugar | RAW BROWN SUGAR | 3.20 | 3.57 |
| 36 | Sugar | SUGAR | 3.92 | 4.00 |
| 37 | Vegetables | PUMPKIN | 1.17 | 1.17 |
| 38 | Vegetables | AMARANTH | 0.94 | 1.27 |
| 39 | Vegetables | JUTE LEAVES | 1.51 | 1.57 |
| 40 | Vegetables | CUCUMBER | 0.98 | 0.95 |
| 41 | Vegetables | BOTTLE GOURD | 0.69 | 0.99 |
| 42 | Vegetables | BITTER GOURD | 2.47 | 2.39 |
| 43 | Vegetables | SPONGE GROUD | 1.13 | 0.97 |
| 44 | Vegetables | POINTED GOURD | 2.88 | 2.93 |
| 45 | Vegetables | BEANS | 1.70 | 2.82 |
| 46 | Vegetables | ONION | 2.07 | 2.82 |
| 47 | Vegetables | GARLIC | 5.06 | 4.57 |
| 48 | Vegetables | OKRA | 1.47 | 1.06 |
| 49 | Vegetables | TOMATO | 1.84 | 4.24 |
| 50 | Vegetables | DRUMSTICK | 3.04 | 5.05 |
| 51 | Vegetables | EGGPLANT | 1.60 | 1.67 |
| 52 | Fruits | APPLE | 6.84 | 6.00 |
| 53 | Fruits | BANANA | 1.75 | 2.21 |
| 54 | Vegetables | CABBAGE | 1.59 | 2.16 |
| 55 | Vegetables | GREEN CHILIES | 3.58 | 5.7 |
| 56 | Condiment vegetables | FLAX SEED | 2.96 | 4.37 |
| 57 | Condiment vegetables | FIVE SPICES | 11.72 | 14.58 |
| 58 | Condiment vegetables | FENUGREEK | 5.41 | 8.43 |
| 59 | Condiment vegetables | YELLOW MUSTARD | 5.39 | 8.06 |
| 60 | Fats | BUFFALO GHEE | 28.6 | 35.2 |
| 61 | Fats | COW GHEE | 46.3 | 59.2 |
| 62 | Fats | VEGETABLE GHEE | 6.15 | 10.52 |
| 63 | Snacks | BEATEN RICE, SNACKS | 2.13 | 2.85 |
| 64 | Dairy | CURD | 3.2 | 4.16 |

***Prices are given per 100 g purchased food.**

# Web Table 2 Characteristics of wealth groups in Dhanusha


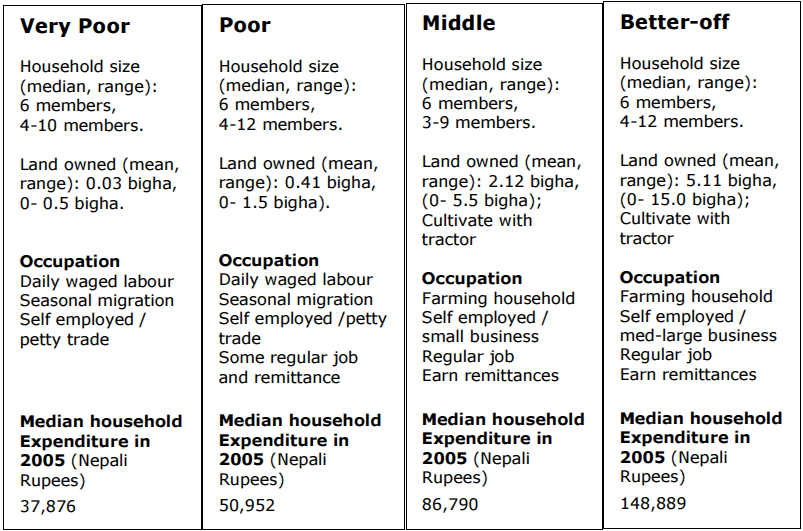


Bigha = ~6,772.63 m² or 0.677 hectares

# Web Table 3 Nutritional requirements of individual household members

|  |  | Energy  (Kcal) | Fat  (g) | Calcium  (mg) | Magnesium  (mg) | Zinc  (mg) | Iron  (mg) | Vitamin B1  (mg) | Vitamin B2  (mg) | Niacin EQ^1^  (mg) | Vitamin B6  (mg) | Pantothenic acid  (mg) | Folic acid  (μg) | Vitamin B12  (μg) | Vitamin C  (mg) | Retinol RE  μg |
| --- | --- | --- | --- | --- | --- | --- | --- | --- | --- | --- | --- | --- | --- | --- | --- | --- |
| 1 | Male 2-3 years | 1125 | 38 | 500 | 60 | 4.1 | 5.8 | 0.5 | 0.5 | 6 | 0.5 | 2 | 150 | 0.9 | 30 | 400 |
| 2 | Male 5-6 years | 1475 | 49 | 600 | 76 | 4.8 | 6.3 | 0.6 | 0.6 | 8 | 0.6 | 3 | 200 | 1.2 | 30 | 450 |
| 3 | Female 13-14 years | 2375 | 79 | 1300 | 220 | 7.2 | 14 | 1.1 | 1 | 16 | 1.2 | 5 | 400 | 2.4 | 40 | 600 |
| 4 | Man, 30-59y, 50 kg, moderately active | 2750 | 92 | 1000 | 260 | 7 | 13.7 | 1.2 | 1.3 | 16 | 1.3 | 5 | 400 | 2.4 | 45 | 600 |
| 5 | Woman, 18-29y, 45 kg, moderately active | 2200 | 73 | 1000 | 220 | 4.9 | 29.4 | 1.1 | 1.1 | 14 | 1.3 | 5 | 400 | 2.4 | 45 | 500 |
| 6 | Woman, 30-59y, 45 kg, lightly active | 1950 | 65 | 1000 | 220 | 4.9 | 29.4 | 1.1 | 1.1 | 14 | 1.3 | 5 | 400 | 2.4 | 45 | 500 |

**Web Table 4 Daily household^1^ requirements for energy and nutrients and percentage met by the minimum-cost, nutritionally adequate diet generated using linear programming, for Dhanusha in 2005 and 2008**

| Nutrients | Household Requirement | % requirements met by nutritionally adequate diet, 2005 | % requirements met by nutritionally adequate diet, 2008 |
| --- | --- | --- | --- |
| Energy (kcal) | 11875 | 100.0 | 100.0 |
| Protein (g) | 186.0 | 168.1 | 170.3 |
| Fat (g) | 395.8 | 100.0 | 100.0 |
| Vitamin A (μg RAE) | 3050.0 | 100.0 | 100.0 |
| Thiamine (mg) | 5.6 | 154.7 | 153.6 |
| Riboflavin (mg) | 5.6 | 156.0 | 181.7 |
| Vitamin B6 (mg) | 6.2 | 150.3 | 165.8 |
| Vitamin B12 (μg) | 11.7 | 100.0 | 104.3 |
| Vitamin C (mg) | 235.0 | 318.8 | 220.7 |
| Calcium, absorbed (mg) | 1620 | 100.0 | 100.0 |
| Magnesium (mg) | 1056 | 167.5 | 148.7 |
| Iron, absorbed (mg) | 9.9 | 100.0 | 100.0 |
| Folic acid (μg) | 1950.0 | 208.8 | 208.8 |
| Zinc (mg) | 32.9 | 199.0 | 186.0 |

^1^Typical household with 6 members: Boy (2-3 years), Boy (5-6 years), Adolescent girl (13-14 years),

Adult male (37 years), Adult female (28 years), Adult female (45-50 years).

# Web table 5 Cash-income sources by data collection sites and sources

| **Every VDC** | **12 purposively selected VDC** | **Government offices** |
| --- | --- | --- |
|  |  |  |
| **Daily Waged labour categories** | **Daily Waged labour categories** | **District Development Committee** |
|  |  |  |
| Agricultural labour | Digging irrigation canals | Support staff |
|  | Road repair | Junior Administrative clerk |
|  | House making |  |
|  | Thatching roof |  |
| **Migratory labour to** | Rickshaw driving |  |
| Janakpur | Borehole digging |  |
| Kathmandu | Construction labour |  |
| India | Carpenter |  |
| Other places | Skilled factory labour |  |
|  | Unskilled factory labour |  |
|  | Wedding band play |  |
|  |  |  |
| **Remittances from** | **Self-employment/ trade** | **District Public Health Office** |
|  |  |  |
| Arab | Small tea shop | Maternal and Child Health |
| Malaysia | Small snack shop | Worker/ Village Heath worker/ |
| Others | Small restaurant / hotel | Auxiliary Nurse Midwife/ |
|  | Small vegetable stall | Auxiliary Health worker/ |
|  | Mobile vegetable stall | Community Medical Assistant |
|  | Medium vegetable stall | Health Assistant/ Staff |
|  | Small grocery shop | Nurse / BA Nurse |
|  | Small alcohol shop | Doctor |
|  | Medium grocery shop | District Education Office |
|  | Small medicine shop | Primary school teacher |
|  | Medium medicine shop | Secondary school teacher |
|  | Small medicine shop | Campus lecturer |
|  | Small cloth shop |  |
|  | Livestock middle man |  |
|  | Large cloth shop |  |
|  | Large wholesale shop |  |
|  |  |  |
